# Supplementary material for: Overexpression of Extradomain-B Fibronectin is Associated with Invasion of Breast Cancer Cells
Source: Cells. 2020 Aug 3;9(8):1826. doi: 10.3390/cells9081826 (PMC7463489; doi:10.3390/cells9081826)
Supplement: Supplementary file 1 [file cells-09-01826-s001.pdf]

Supplementary Figure 1

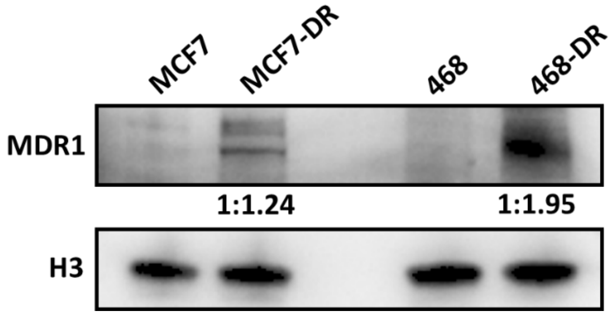

Fig S1. Western blot analysis shows significant upregulation of the drug resistance marker P-glycoprotein 1 [multidrug resistance protein 1 (MDR1)] in MCF7-DR and MDA-MB-468-DR cells over their respective counterparts. Histone H3 was used as loading control. The western blot band intensities were normalized to those of histone H3 bands in FIJI, and the protein level changes are expressed as ratio of resistant over non-resistant cells, below the respective lanes.

Supplementary Figure 2

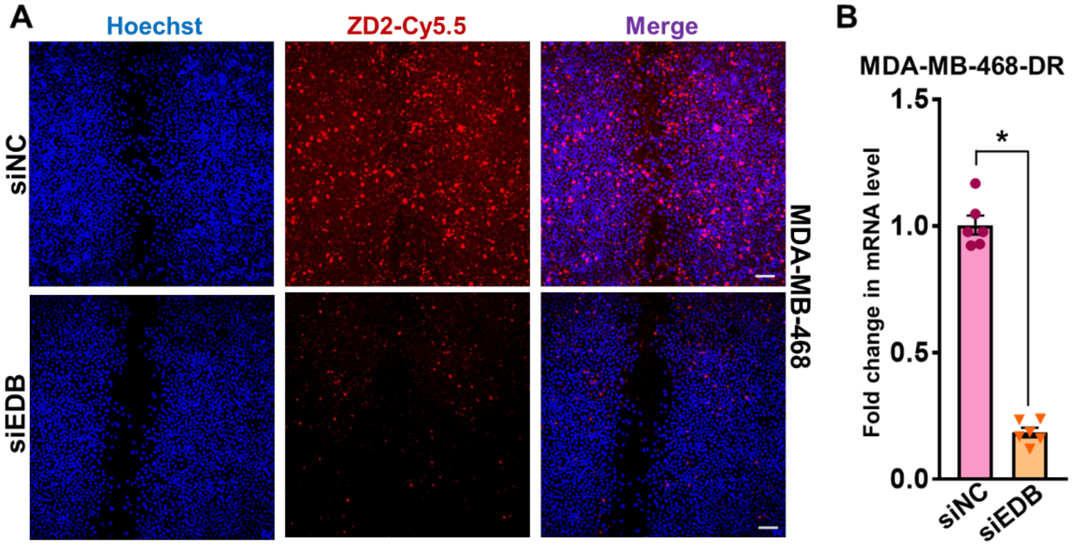

Fig S2. RNAi of EDB-FN using ECO/siEDB nanoparticles abolishes **A.** ZD2-Cy5.5 binding to EDB-FN and **B.** significantly decreases EDB-FN mRNA level, confirming EDB-FN-specific binding of ZD2-Cy5.5 and EDB-FN silencing, respectively. ECO/siLuc nanoparticles were used as negative control. Bars denote mean ± sem and dots denote independent replicates. *$p<0.05$ using Mann-Whitney U test. Scale bar = 100 µm.

Western blot details:

Samples were run on 4-20% SDS-PAGE gels. MagicMark (Life Technologies, https://www.thermofisher.com/order/catalog/product/LC5602#/LC5602) was used as ladder (picture provided below). After transferring proteins from gel to nitrocellulose membranes, blocking was performed in milk. The membranes were cut vertically or horizontally to probe for different sized proteins in BSA-TBS-T-diluted primary antibodies. After secondary antibody incubation, the membranes were developed with Signal Fire PLUS ECL solution (Cell Signaling Technology).

GelDoc XRS+ Imager (Biorad) based on CCD high-resolution, high-sensitivity detection technology, was used to develop the membranes. This machine provides the option to set up multiple exposures for multiple proteins in a single protocol. Because of this, the uncropped images provided below may contain more than 1 membrane with its own protein. The 2 membranes (proteins) will have their own optimal exposure times. Similarly, in different cell lines, the same protein would need its own optimal exposure time. Both protein labels/exposure times are provided for transparency; however, the labels marked in red denote the specific protein/samples depicted in the manuscript and are noted in the individual captions.

Since the orientation of the gel/membrane does not matter during exposure, the MagicMark ladder has been used to orient the order of the samples. The presence of the ladder will determine lane and orientation (eg. MagicMark>>Blank lane>>Lane 1>>) OR (<<Lane 1<<Blank lane<<MagicMark).

Two methods were used for normalization. After protein extraction, Lowry assay was performed to assess total protein concentration. Using these values, equal amount of protein (40 µg) was loaded in each well. This equal loading was validated by using actin as loading control. Since molecular weight of Actin is 45 kD and other desired proteins also have the same molecular weight (AKT-60 kD, SRp55-55 kD, Slug-35 kD), each membrane could not be probed for actin. However, at least two independent membranes were probed with actin with other proteins that are farther away on the ladder (Ecad-135kD, N-cad-145kD).

All the blots were run and probed fresh and once. Blots were not stripped for re-probing with other antibodies because we found that the stripping buffer also striped off the proteins from the membranes.

Figure 1 - Sharper bands and more sensitive detection with the MagicMark™ XP Standard using various western detection systems

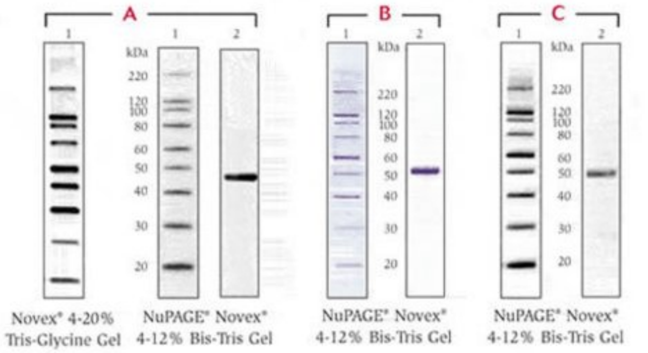

Lane 1: 5 µl MagicMark™ XP Standard
Lane 2: 2 ng of protein (Note: the 4-20% Tris-Glycine gel does not contain a lane 2)

MagicMark™ XP Standard and an expressed protein containing a V5 epitope tag were separated on the indicated gel and transferred onto a nitrocellulose membrane. The blots were probed with a 1:5,000 dilution of mouse anti-V5 primary antibody and detected with either an alkaline phosphatase-conjugated anti-mouse secondary antibody using Invitrogen's WesternBreeze® Chemiluminescent Kit (A) and Chromogenic Kit (B), or a horseradish peroxidase conjugated anti-mouse secondary antibody using another manufacturer's luminol substrate (C). The results show that the same reagents and protocol used for the target protein allow sensitive detection of MagicMark™ XP protein bands directly on blots using various western detection systems.

Figure 2C

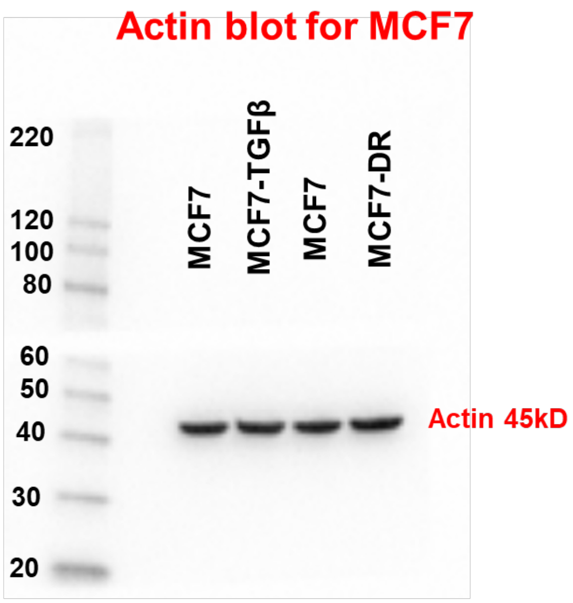

Actin expression in MCF7, MCF7-TGF-β, and MCF7-DR cells.

Figure 2D

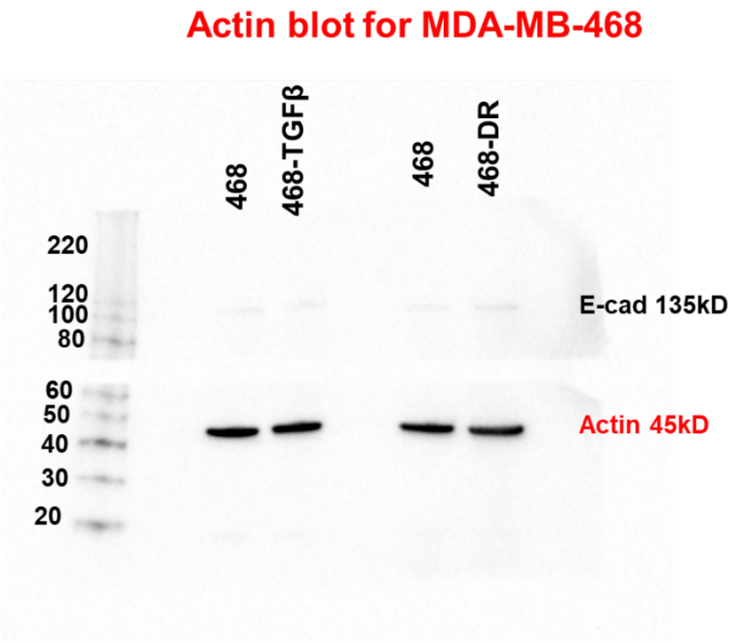

Actin expression in MDA-MB-468, MDA-MB-468-TGF-β, and MDA-MB-468-DR cells.

Figure 4A

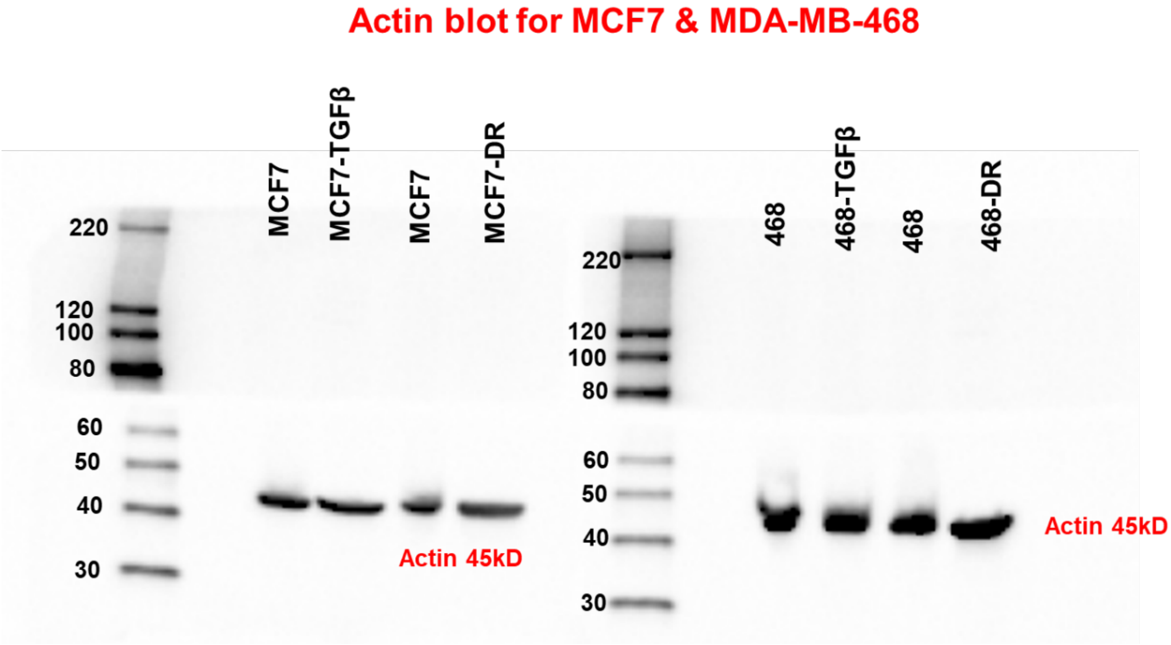

Actin expression in MCF7 and MDA-MB-468 cells, and their invasive derivatives.

Figure 2C and 2D

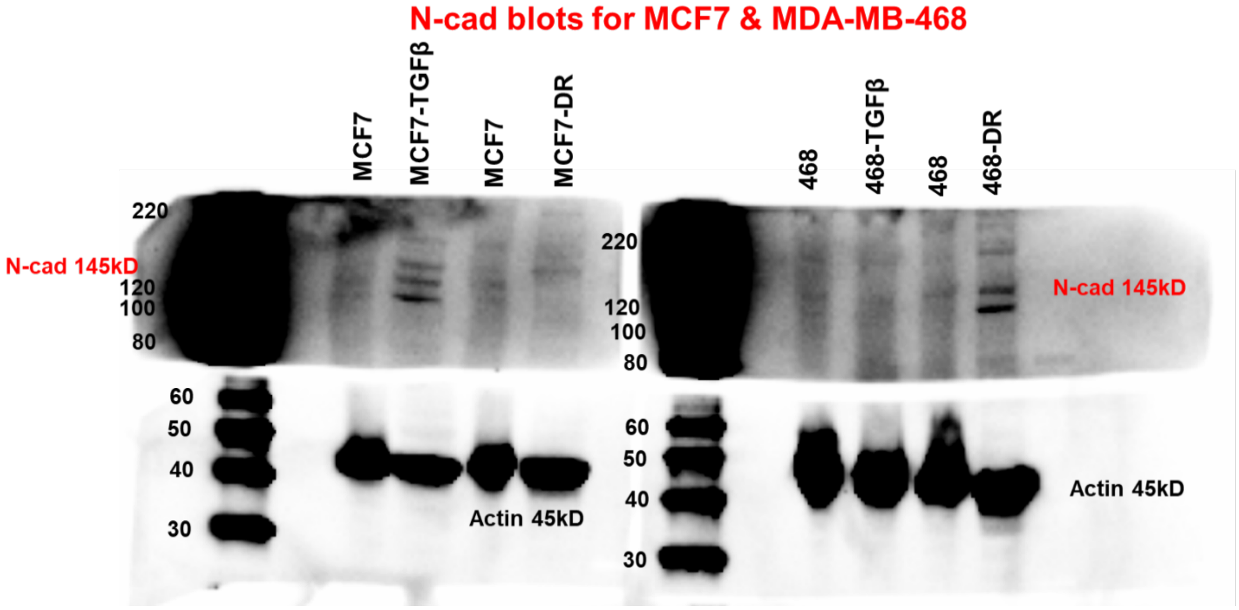

Comparison of **N-cad** expression in **Fig. 3A** MCF7 and **Fig. 3B** MDA-MB-468 cells, with their invasive derivatives.

Figure 2D

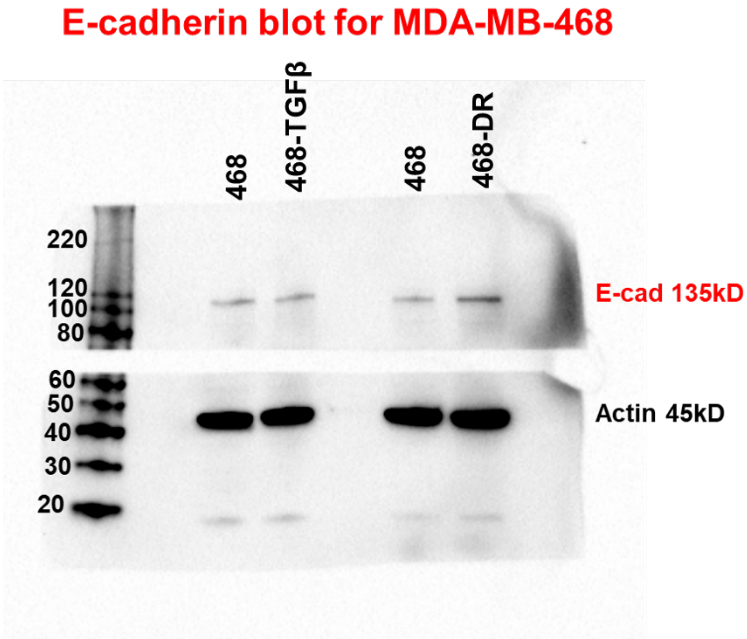

Comparison of **E-cad** expression in MDA-MB-468 cells, with their invasive derivatives.

Figure 4A

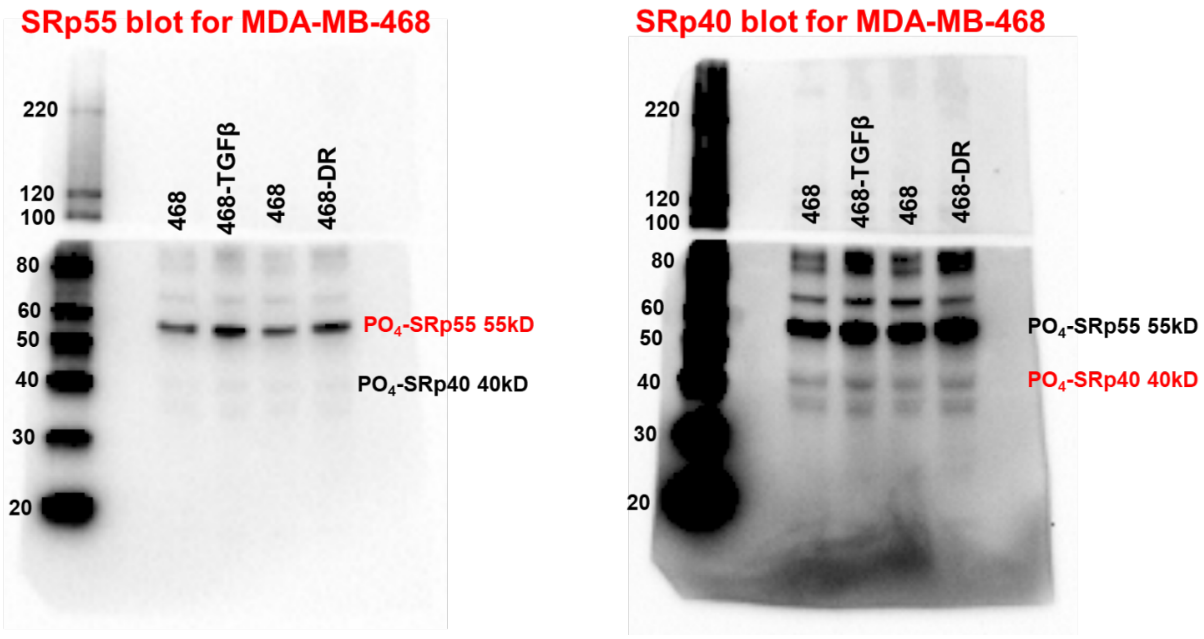

Blot 1. Comparison of **pSRp55** expression in MDA-MB-468 cells, with their invasive derivatives. Blot 2. Comparison of **pSRp40** expression in MDA-MB-468 cells, with their invasive derivatives. Membranes were exposed at 2 time points to visualize bands for the 2 proteins (highlighted in red).

Figures 2C and 4A

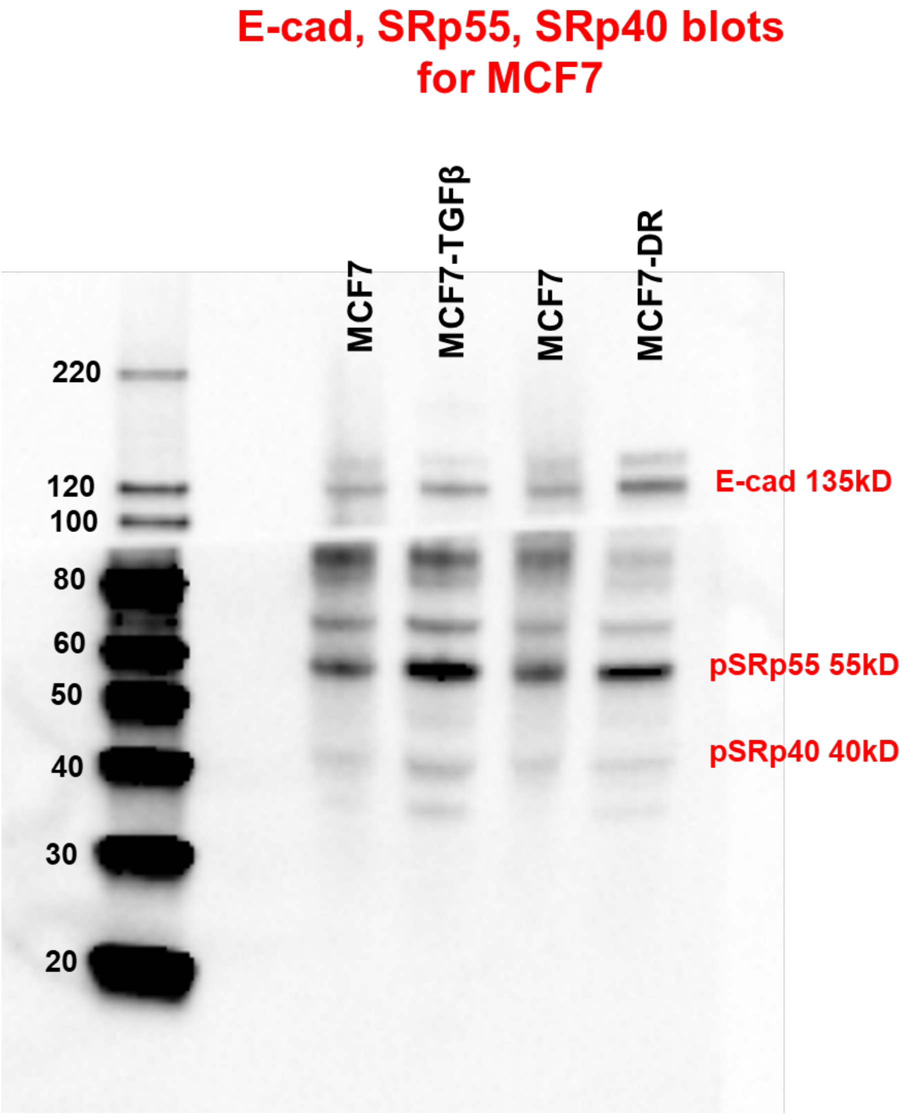

Comparison of **Fig. 2C E-cad** and **Fig 4A. pSRp55** and **pSRp40** expression in MCF7 cells, with their invasive derivatives.

Figure 2D

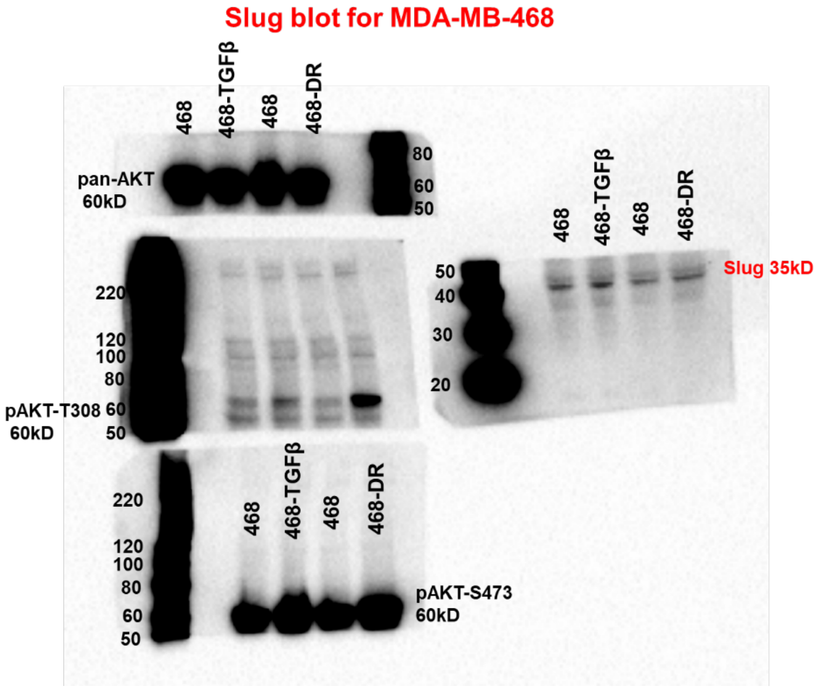

Comparison of **Slug** expression in MDA-MB-468 cells, with their invasive derivatives.

Figure 4A

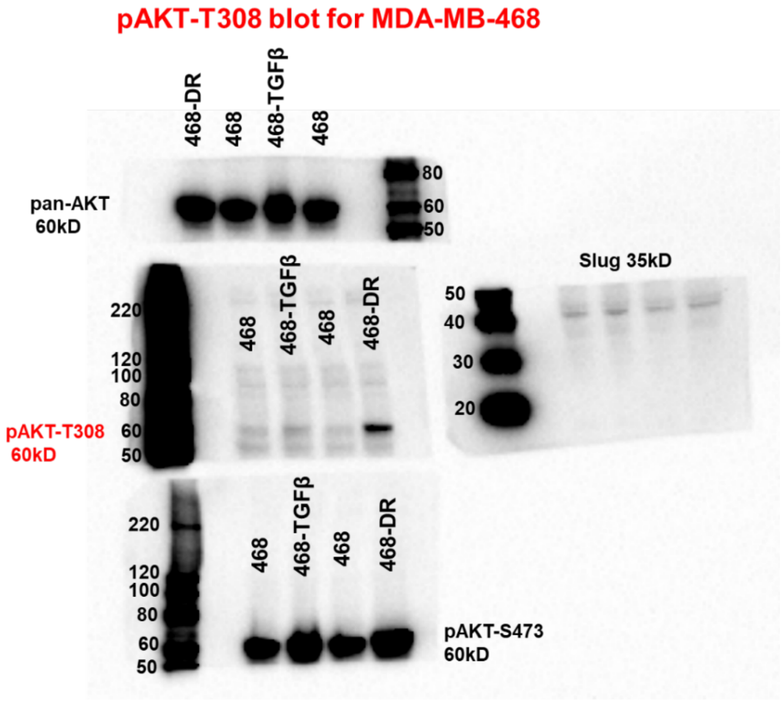

Comparison of **pAKT-T308** expression in MDA-MB-468 cells, with their invasive derivatives.

Figure 4A

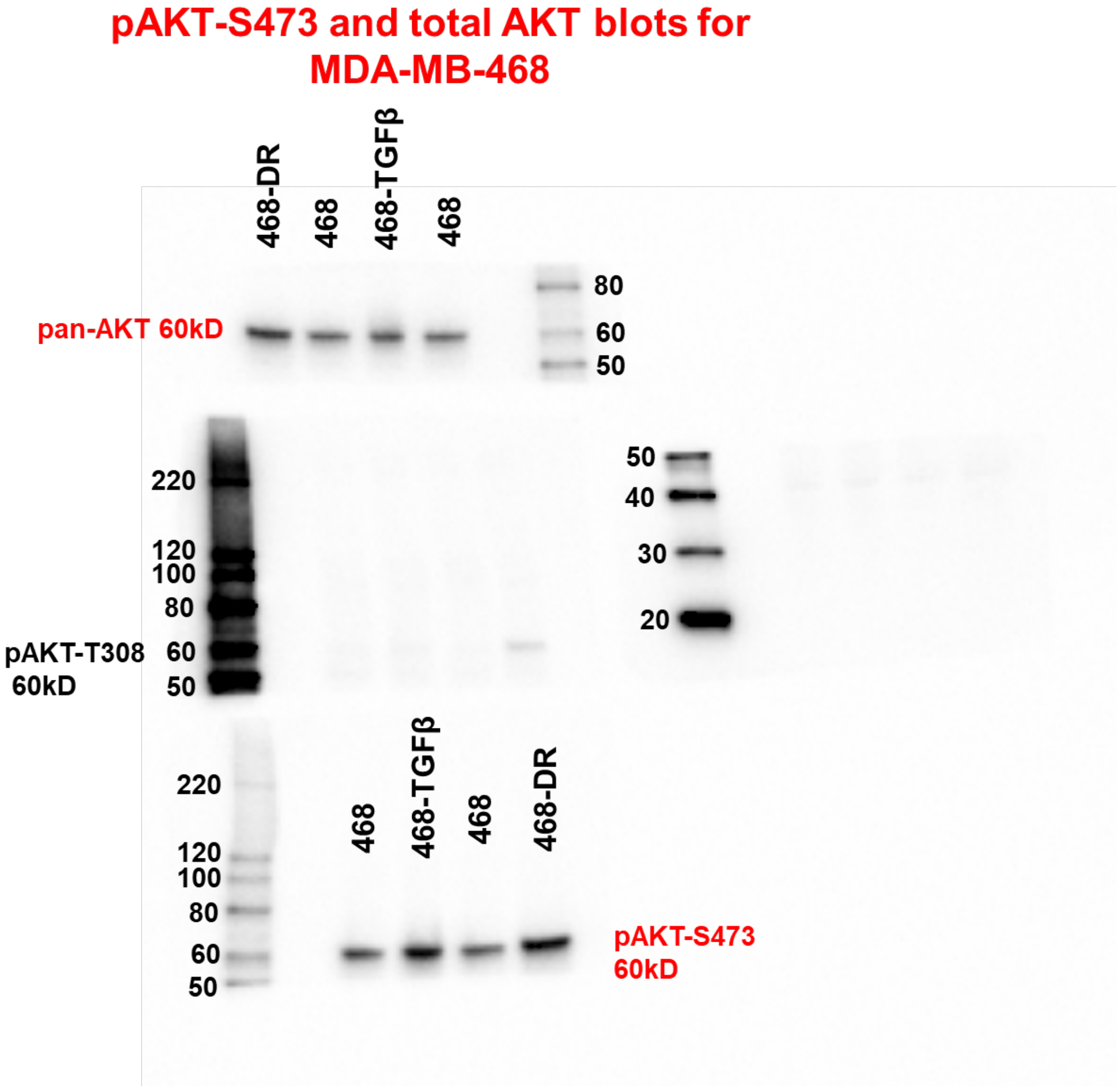

Comparison of **total AKT and pAKT-S473** expression in MDA-MB-468 cells, with their invasive derivatives. The membranes were exposed for at least 3 different time points to visualize multiple proteins (highlighted in red on this page and previous page).

Figure 2C

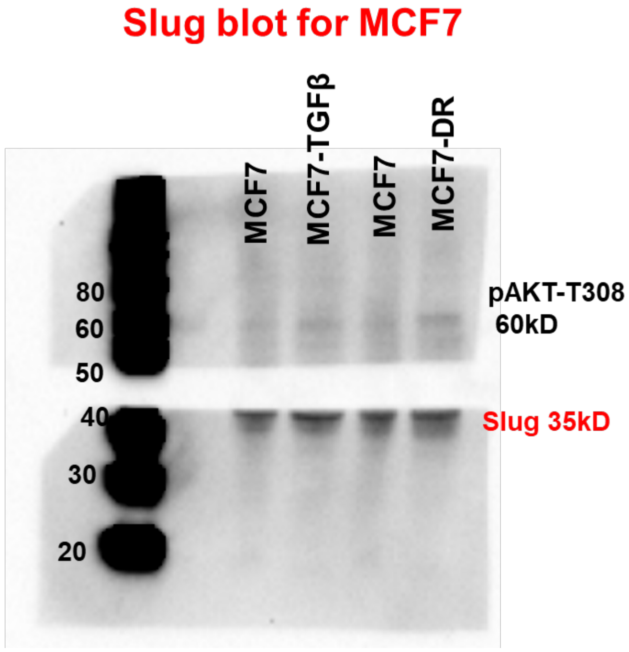

Comparison of **Slug** expression in MCF7 cells, with their invasive derivatives.

Figure 4A

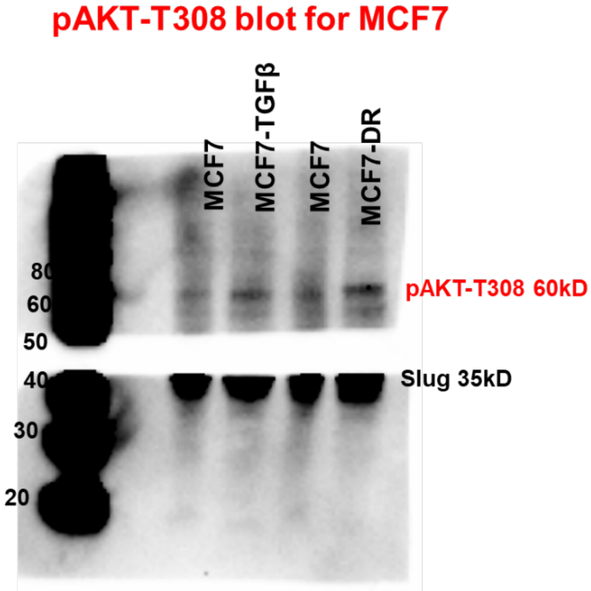

Comparison of **pAKT-T308** expression in MCF7 cells, with their invasive derivatives.

Same membranes exposed at 2 different time points to visualize 2 proteins (in red).

Figure 4A

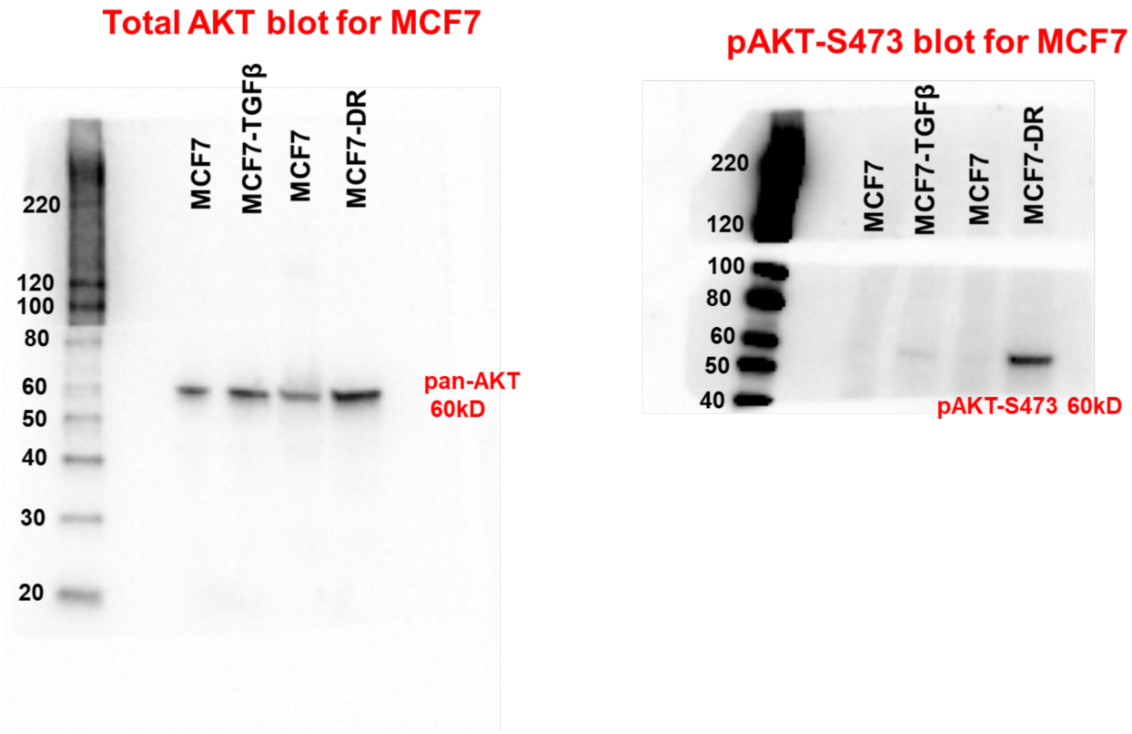

Comparison of **total AKT and pAKT-S473** expression in MCF7 cells, with their invasive derivatives.

Figure 4B

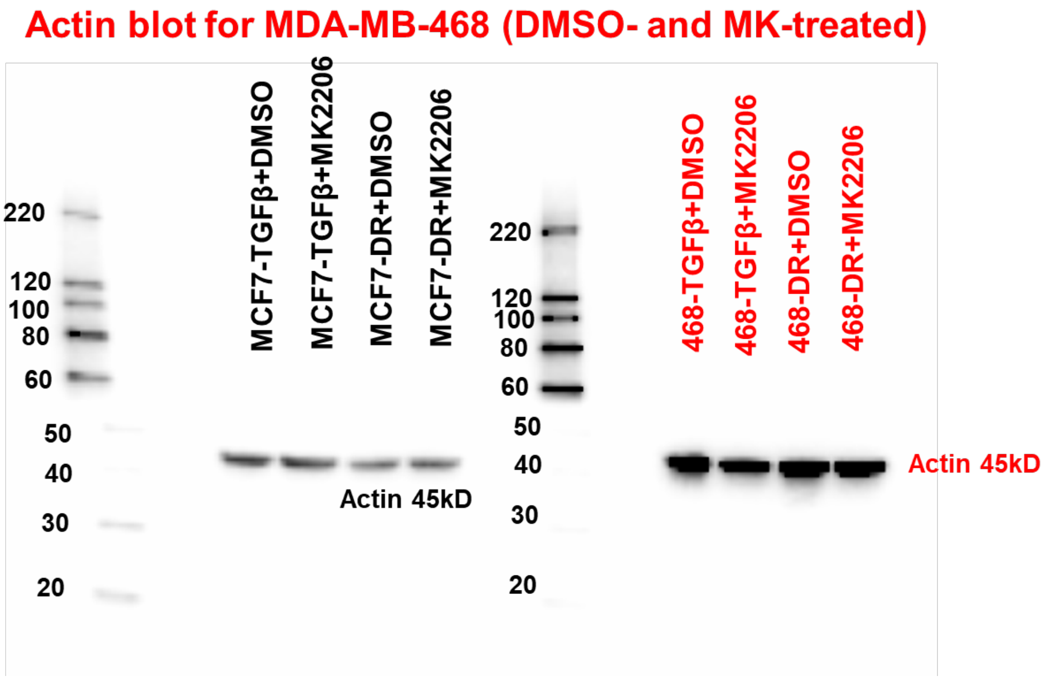

Expression of **Actin** expression in DMSO- and MK2206-treated MDA-MB-468-TGFβ and MDA-MB-468-DR cells (highlighted in red).

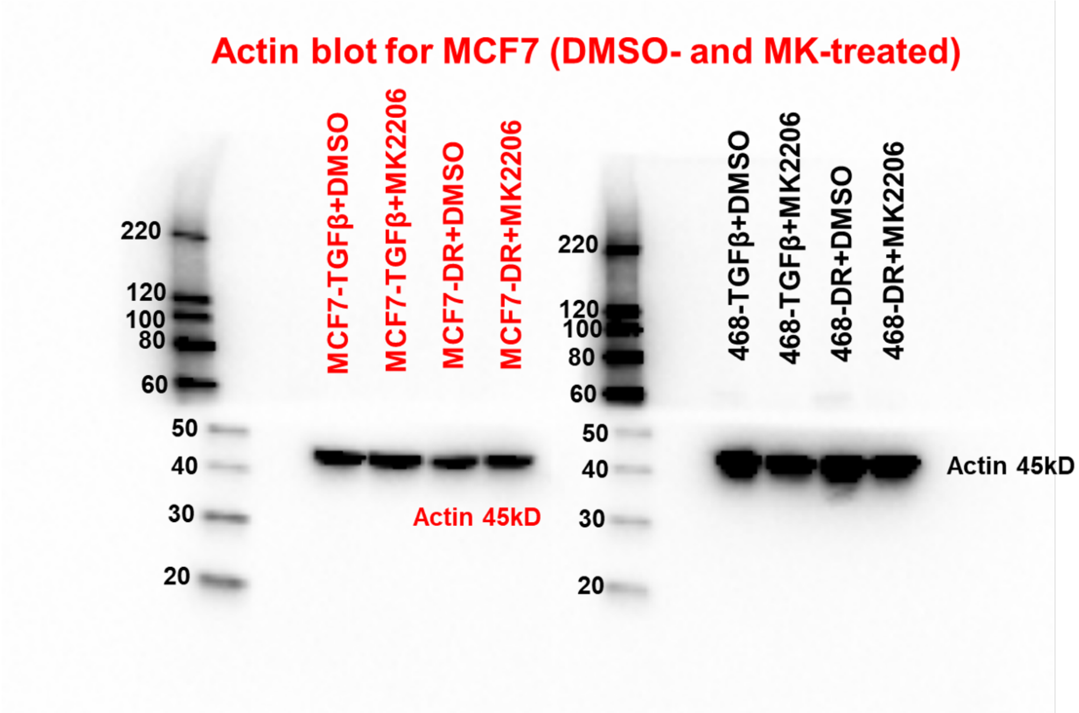

Expression of **Actin** expression in DMSO- and MK2206-treated MCF7-TGFβ and MCF7-DR cells (highlighted in red). Same membrane was exposed for longer to visualize bands in the MCF7 cells.

Figure 4B

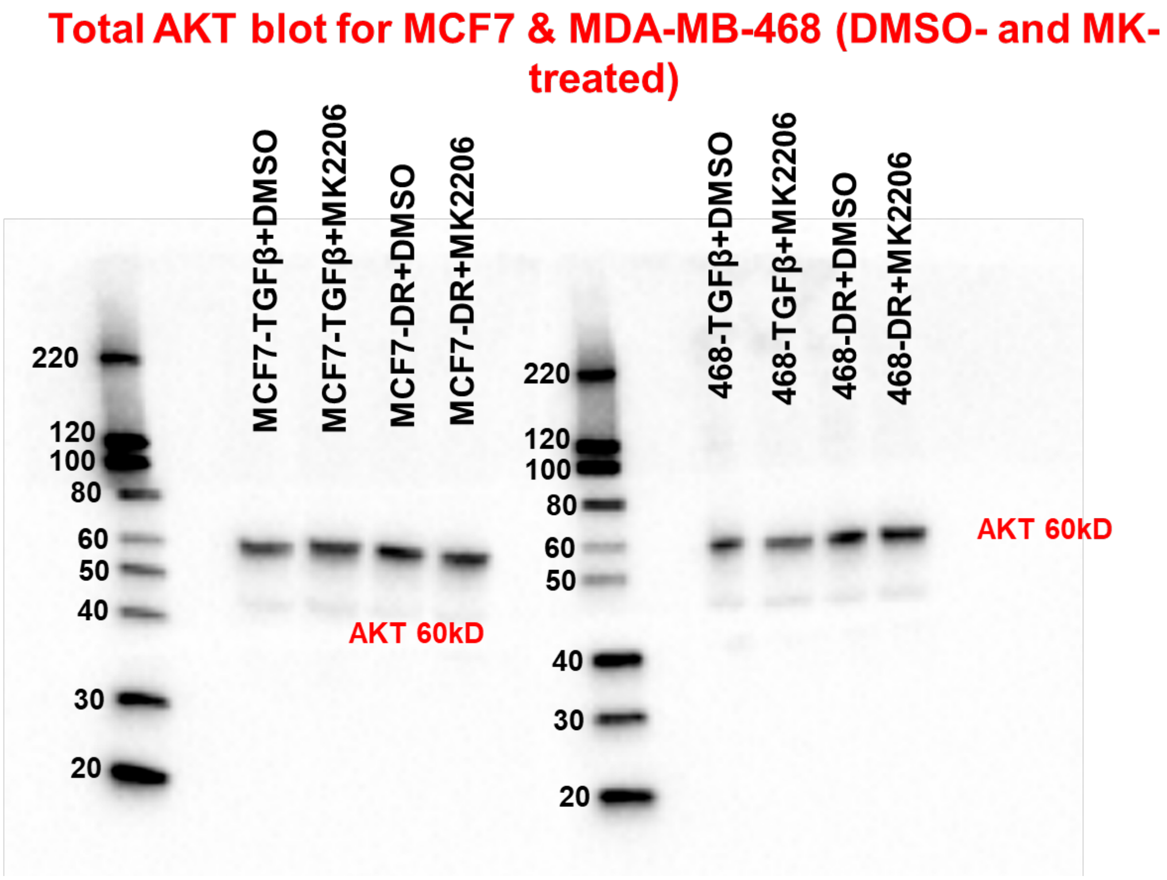

Comparison of **total AKT** expression in DMSO- and MK2206-treated MCF7-TGFβ, MCF7-DR, MDA-MB-468-TGFβ, and MDA-MB-468-DR cells.

Figure 4B

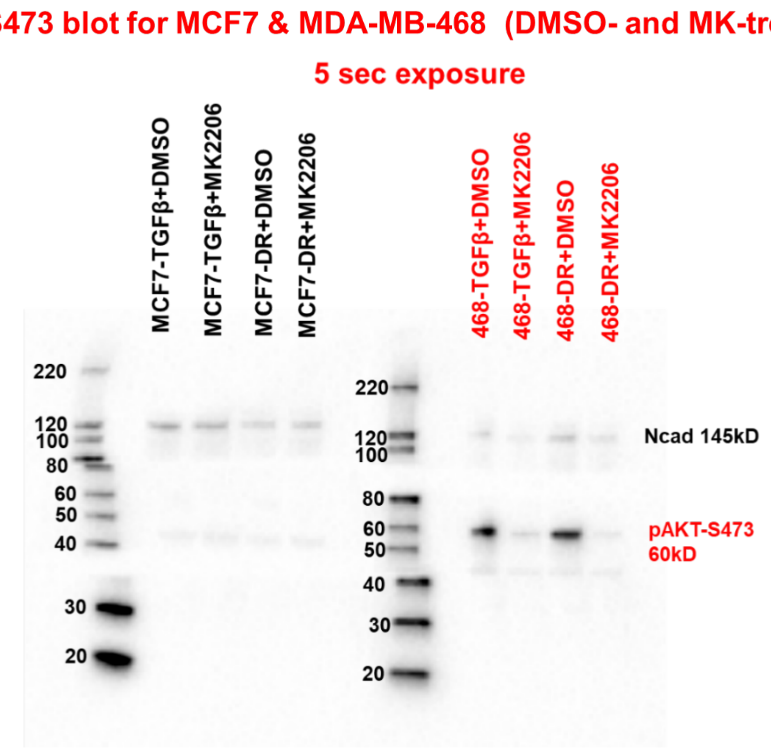

Comparison of **pAKT-S473** expression in DMSO- and MK2206-treated MDA-MB-468-TGFβ and MDA-MB-468-DR cells (highlighted in red). Optimal exposure time was 5 sec for 468 cells.

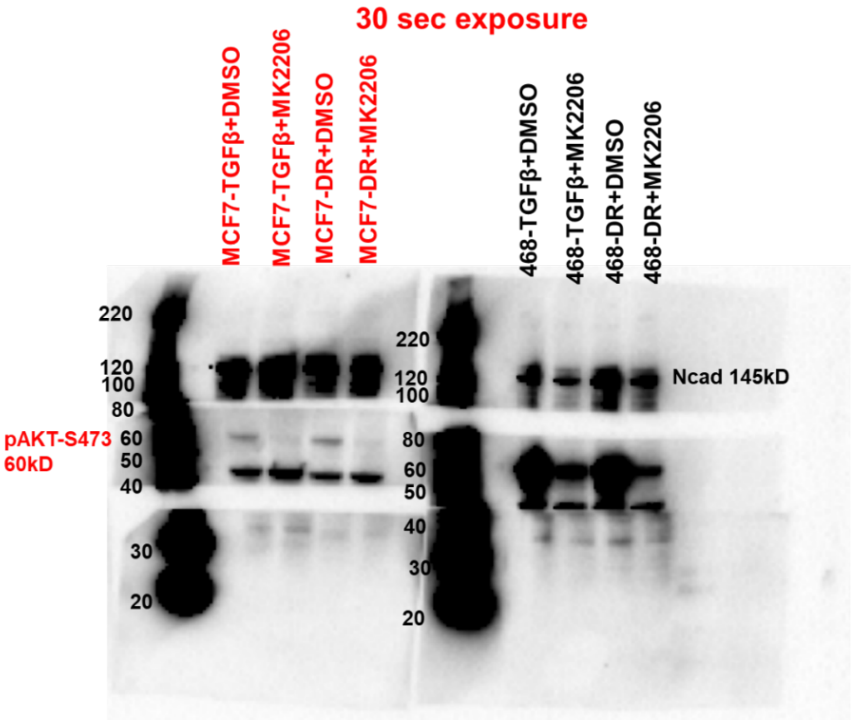

Comparison of **pAKT-S473** expression in DMSO- and MK2206-treated MCF7-TGFβ and MCF7-DR cells (highlighted in red). Same membrane was exposed for longer (30 sec) to visualize bands in the MCF7 cells.

Figure 4B

pAKT-T308 blot for MCF7 & MDA-MB-468 (DMSO- and MK-treated)

10 sec exposure

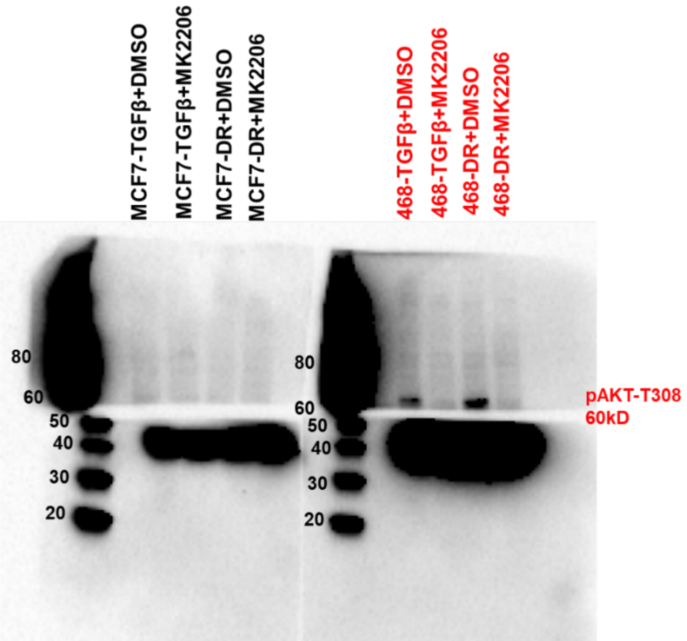

Comparison of **pAKT-T308** expression in DMSO- and MK2206-treated MDA-MB-468-TGFβ and MDA-MB-468-DR cells (highlighted in red). Optimal exposure time was 10 s for 468 cells.

60 sec exposure

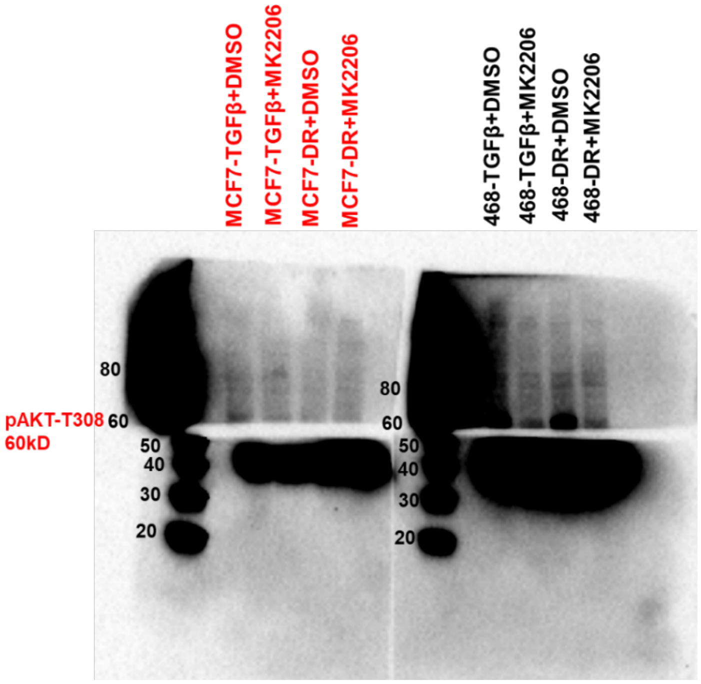

Comparison of **pAKT-T308** expression in DMSO- and MK2206-treated MCF7-TGFβ and MCF7-DR cells (highlighted in red). Same membrane was exposed for longer (60 sec) to visualize bands in the MCF7 cells.

FIGURE 4B

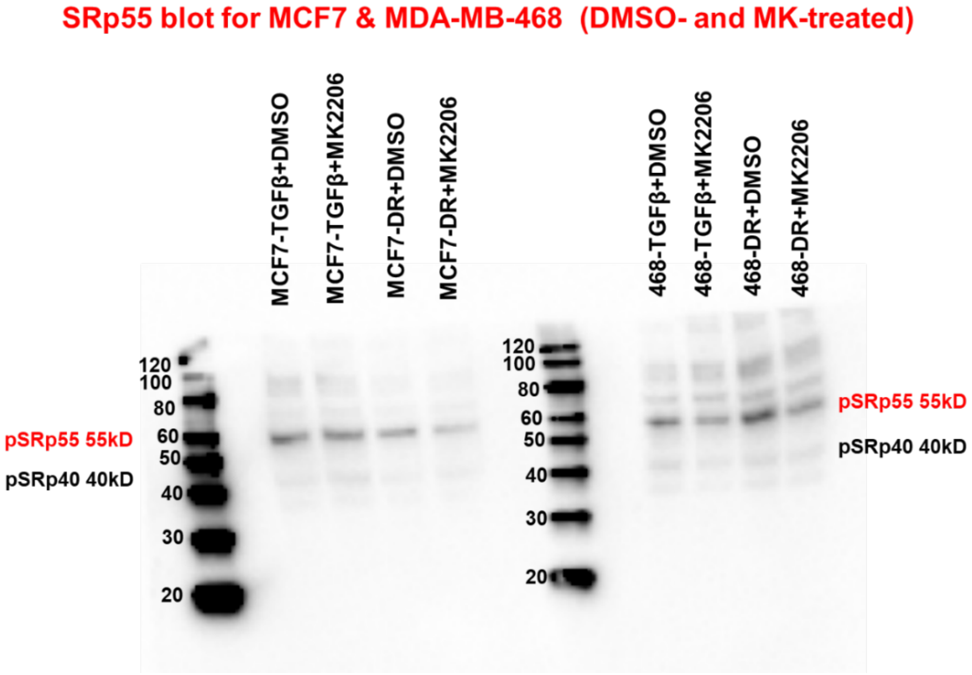

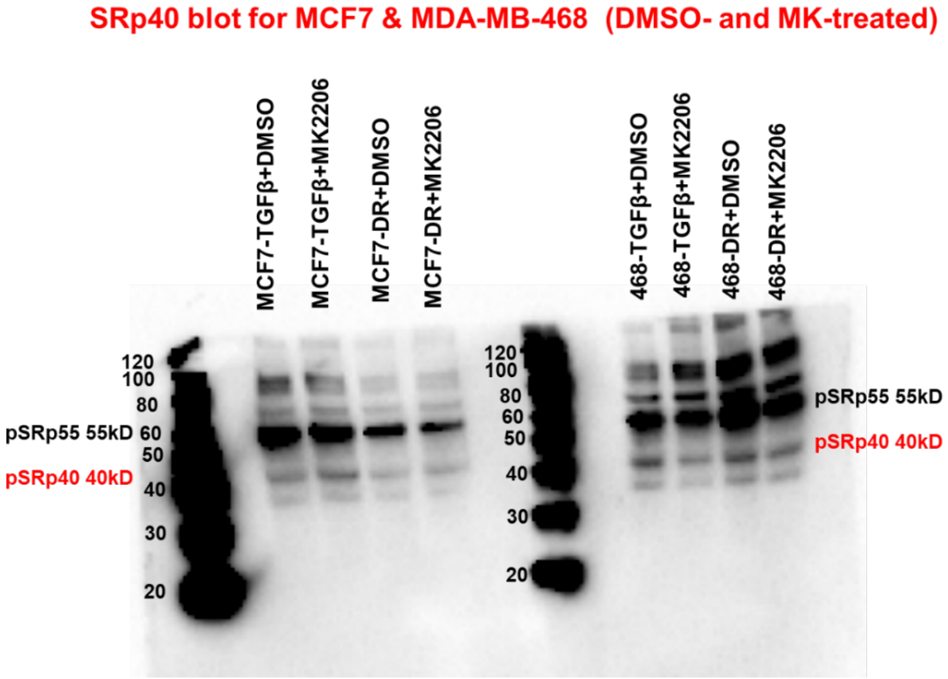

Comparison of **pSRp55 and pSRp40** expression in DMSO- and MK2206-treated MCF7-TGFβ, MCF7-DR cells, MDA-MB-468-TGFβ, and MDA-MB-468-DR cells. Same membrane was exposed for 2 time points to visualize bands for the 2 proteins (highlighted in red)

Supp Fig. 1

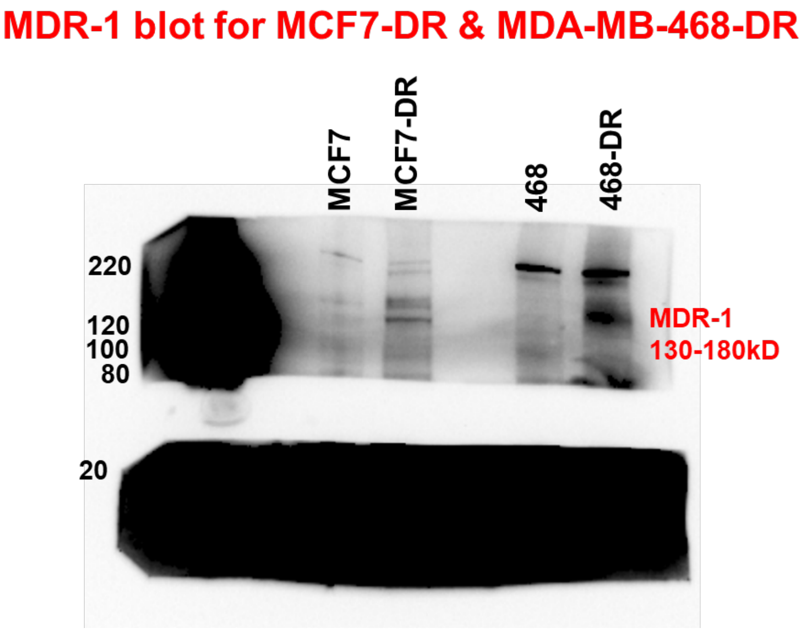

MDR-1 expression in MCF7-DR and MDA-MB-DR cells.

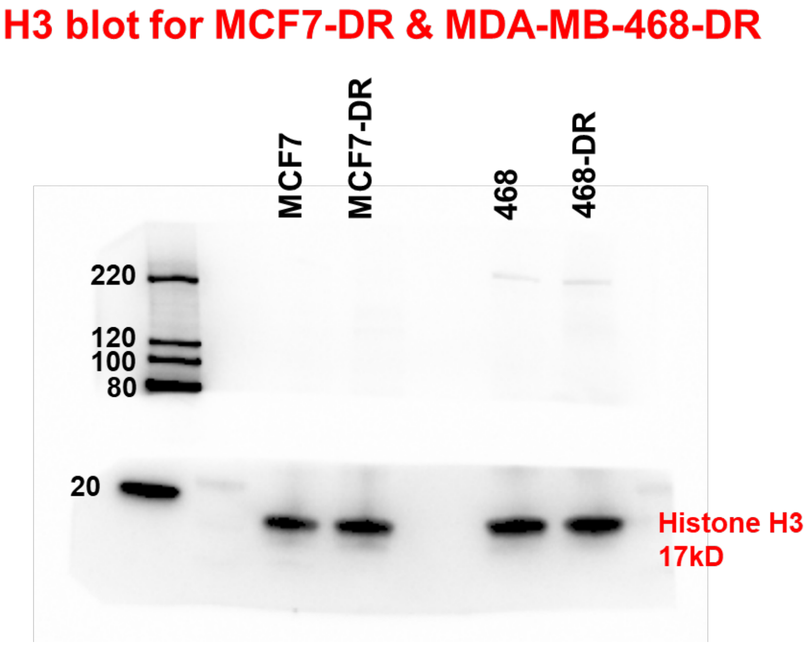

Histone H3 expression in MCF7-DR and MDA-MB-DR cells.

Figure 5A

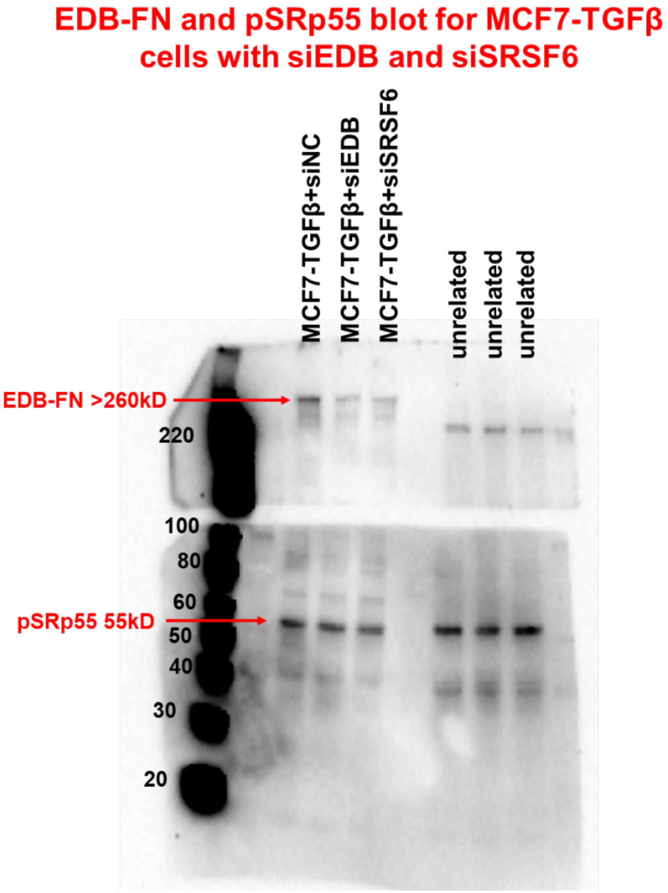

Comparison of **EDB-FN and pSRp55** expression in MCF7-TGFβ cells transfected with siNC, siEDB-FN and siSRSF6 nanoparticles.

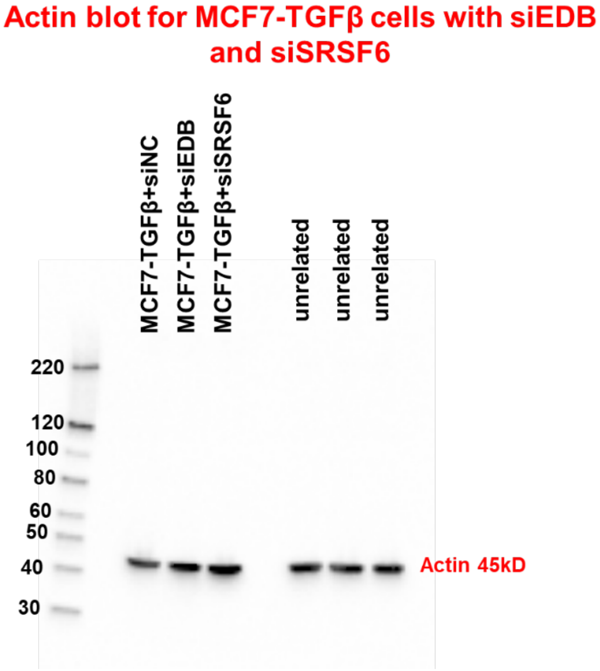

Actin expression in MCF7-TGFβ cells transfected with siNC, siEDB-FN and siSRSF6 nanoparticles.

Figure 5A

EDB-FN and pSRp55 blot for MCF7-DR cells with siEDB and siSRSF6

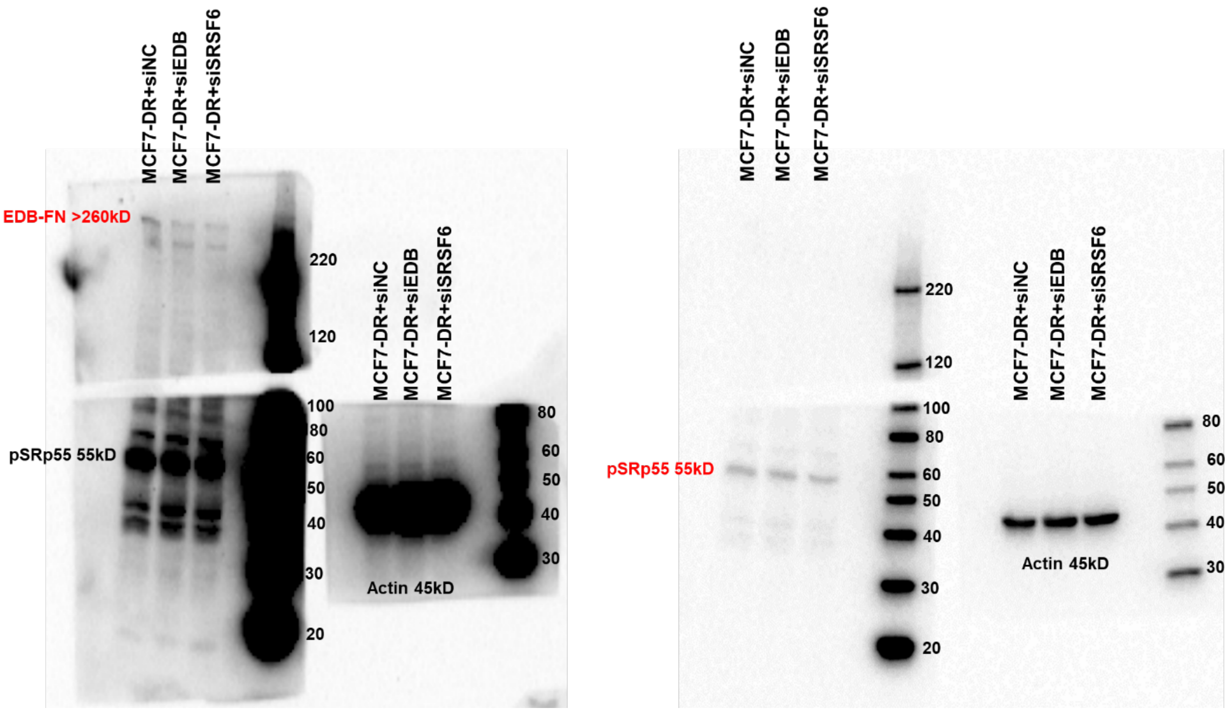

Comparison of **EDB-FN and pSRp55** expression in MCF7-DR cells transfected with siNC, siEDB-FN and siSRSF6 nanoparticles. Same membrane was exposed for 2 time points to visualize bands for the 2 proteins (highlighted in red).

Actin blot for MCF7-DR cells with siEDB and siSRSF6

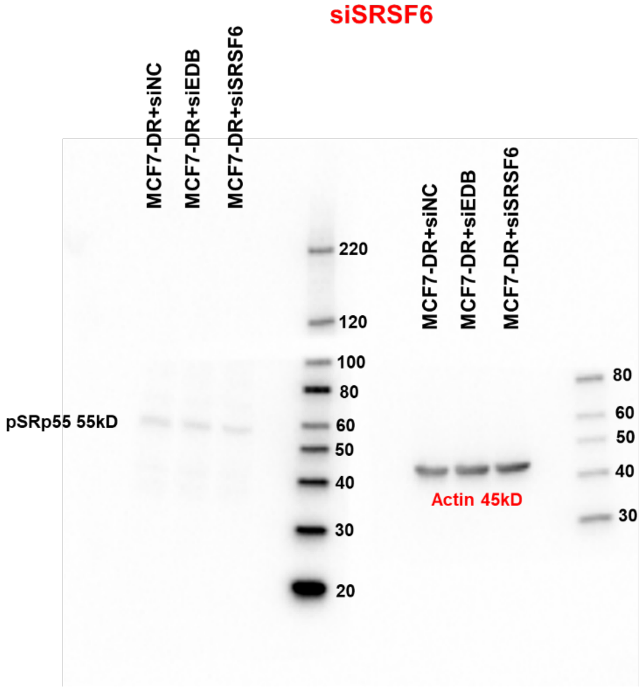

Actin expression in MCF7-DR cells transfected with siNC, siEDB-FN and siSRSF6 nanoparticles.

Figure 5A

EDB-FN and pSRp55 blot for 468-TGFβ cells with siEDB and siSRSF6

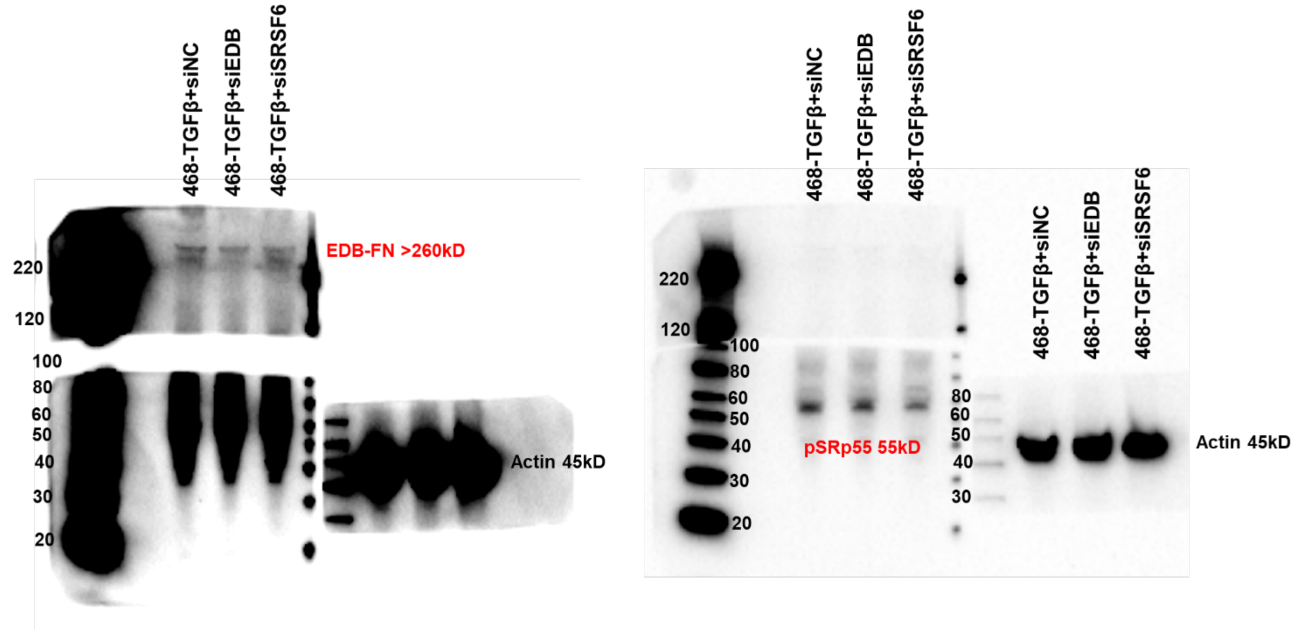

Comparison of **EDB-FN and pSRp55** expression in 468-TGFβ cells transfected with siNC, siEDB-FN and siSRSF6 nanoparticles. Same membrane was exposed for 2 time points to visualize bands for the 2 proteins (highlighted in red).

Actin blot for 468-TGFβ cells with siEDB and siSRSF6

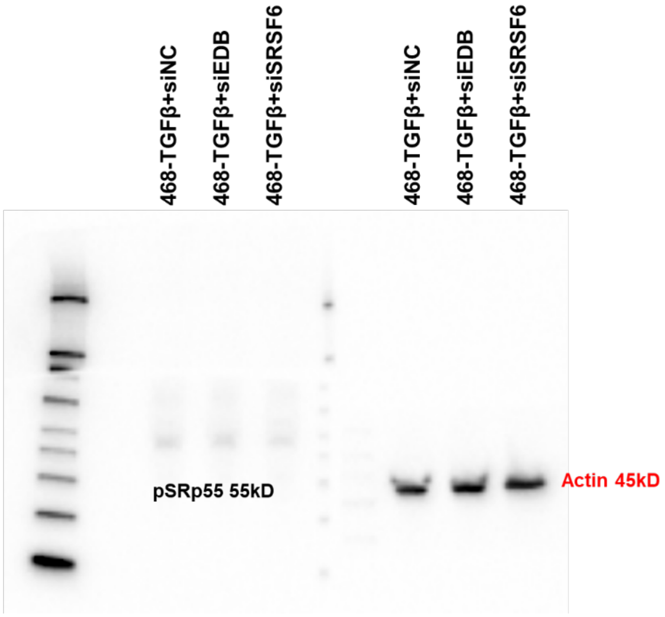

Actin expression in 468-TGFβ cells transfected with siNC, siEDB-FN and siSRSF6 nanoparticles.

Figure 5A

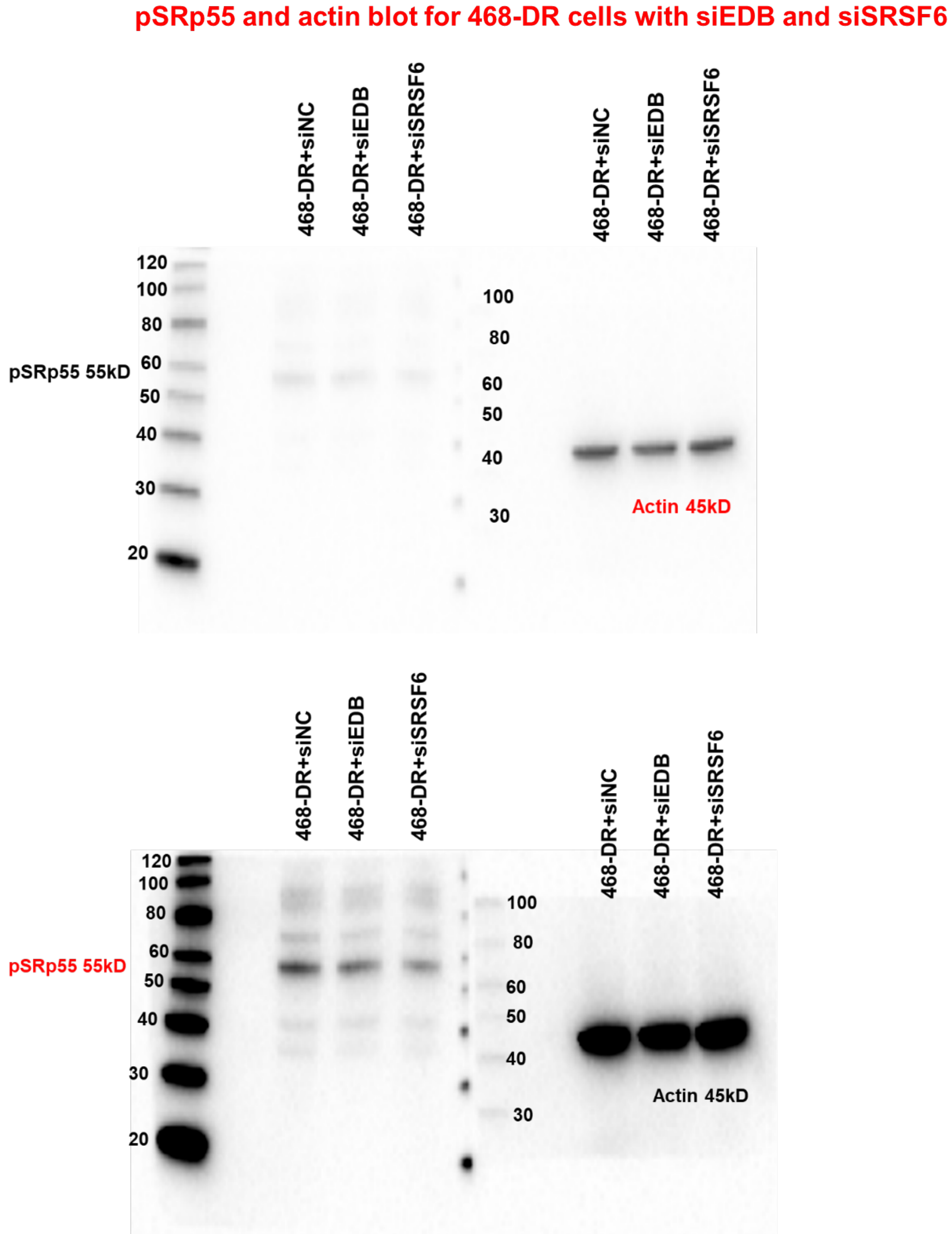

Comparison of **pSRp55 and Actin** expression in 468-DR cells transfected with siNC, siEDB-FN and siSRSF6 nanoparticles. Same membrane was exposed for 2 time points to visualize bands for the 2 proteins (highlighted in red).

Figure 5A

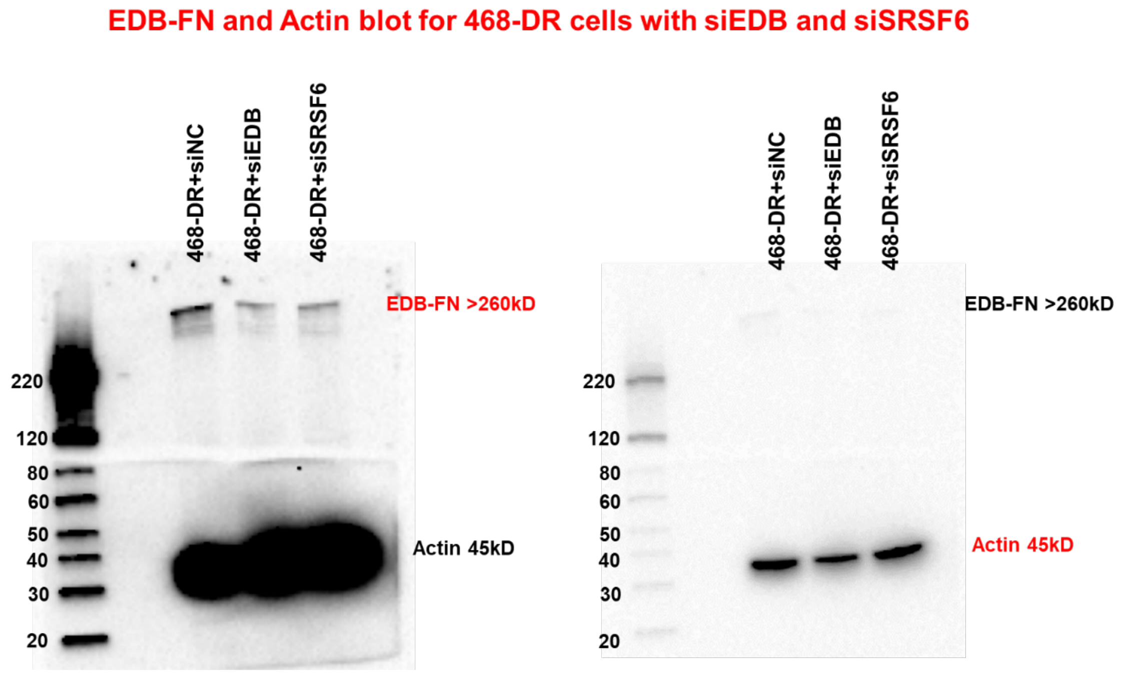

Comparison of **EDB-FN and Actin** expression in 468-DR cells transfected with siNC, siEDB-FN and siSRSF6 nanoparticles. Same membrane was exposed for 2 time points to visualize bands for the 2 proteins (highlighted in red).
